# A Case Report on Carcinomatous Meningitis in a Patient With Double-Hit Lymphoma

**DOI:** 10.7759/cureus.70073

**Published:** 2024-09-24

**Authors:** Gowri Swaminathan, Faateh Rauf, Santino Patrizi, Jonathan Muratori, Debra Ferman

**Affiliations:** 1 Internal Medicine, Icahn School of Medicine at Mount Sinai, Queens Hospital Center, New York, USA; 2 Hematology and Oncology, Icahn School of Medicine at Mount Sinai, Queens Hospital Center, New York, USA

**Keywords:** carcinomatous meningitis, critical care medicine, double-hit lymphoma, high-grade b-cell lymphoma, pleural effusion

## Abstract

A double-hit lymphoma is a high-grade B-cell lymphoma (HGBCL) with *MYC* and *BCL2/BCL6* rearrangements. It is characterized by a rapidly progressive advanced disease, high rates of central nervous involvement (CNS), refractoriness to conventional chemotherapy, and poorer clinical outcomes. Carcinomatous meningitis is a condition in which cancer cells metastasize to the meninges without involving the brain parenchyma; this phenomenon has also been reported in the literature by other terms like “leptomeningeal meningitis,” “leptomeningeal carcinomatosis,” “leptomeningeal metastasis,” or “neoplastic meningitis.” This form of CNS involvement has been described as an infrequent complication with the trajectory of this aggressive lymphoma. We report an illuminating case of a 63-year-old male diagnosed with double-hit lymphoma, which was complicated by fatal carcinomatous meningitis.

## Introduction

High-grade B-cell lymphoma (HGBCL), previously known as double-hit lymphoma, is an aggressive form of non-Hodgkin’s lymphoma (NHL). It is characterized by rearrangements in two specific genes: the MYC gene and BCL2/BCL6, of which BCL2 is the more common gene. HGBCL shares many characteristics with two other B-cell lymphomas - diffuse large B-cell lymphoma (DLBCL) and Burkitt lymphoma [1,2]. About 5% of DLBCLs and about 32-78% of Burkitt lymphomas show similar gene rearrangements as HGBCL and are thus known by the same term; however, prior research has also shown that HGBCL is different from the typical forms of DLBCL and Burkitt lymphoma that do not have the characteristic dual gene rearrangements. It is because of this difference that in 2016, the World Health Organization (WHO) placed HGBCL in a class of its own [3]. Most double-hit lymphomas very commonly have an inferior prognosis and are marked by MYC plus BCL2 rearrangement in about three-fourths of the cases [4].

Leptomeningeal carcinomatosis/carcinomatous meningitis, a term coined by Beerman in 1912, is the metastatic spread of cancer to the pia mater, arachnoid, and subarachnoid space [2,5]. The most common malignancies that can lead to this complication are the lung (9%-25%), breast (5%-8%), melanoma (6%-18%), acute lymphoblastic leukemia (1%-10%), and non-Hodgkin lymphoma (5%-10%) [6-8]. Even with treatment, carcinomatous meningitis has a median survival of up to two to four months [9]; if left untreated, the condition is fatal within four to six weeks [7]. We report our experience with this challenging and aggressive lymphoma to underscore the importance of identifying signs of resistance to conventional chemotherapy earlier during the disease course.

## Case presentation

A 63-year-old male from Jamaica, with no significant past medical history, presented to the emergency room with decreased appetite, a 10-pound weight loss, and generalized weakness for a 1-month duration. Hematology-oncology was consulted after he was noticed to have lumps in the cervical and groin regions. Family history was significant for a brother with colon cancer. The patient was a former smoker of 15 years; he had quit one month ago. He also complained of shortness of breath for a one-month duration. Upon exam, the patient was pale and was found to have three small nodes (Level III and IVa) on the right side of the neck and a small node (Level IVa) on the left side of the neck. Inguinal region palpation revealed lymphadenopathy on the left. The abdominal exam was positive for splenomegaly. A peripheral blood smear exam was positive for a decreased number of leukocytes, questionable Sezary cells, T-cell lymphoma cells, and Burr cells. With pancytopenia in the picture, the possible initial differential diagnoses were adult T-cell leukemia/lymphoma, possibly human T-lymphocyte virus-1 (HTLV-1) associated, other lymphoma, acute leukemia, and myelodysplastic syndrome.

**Figure 1:**
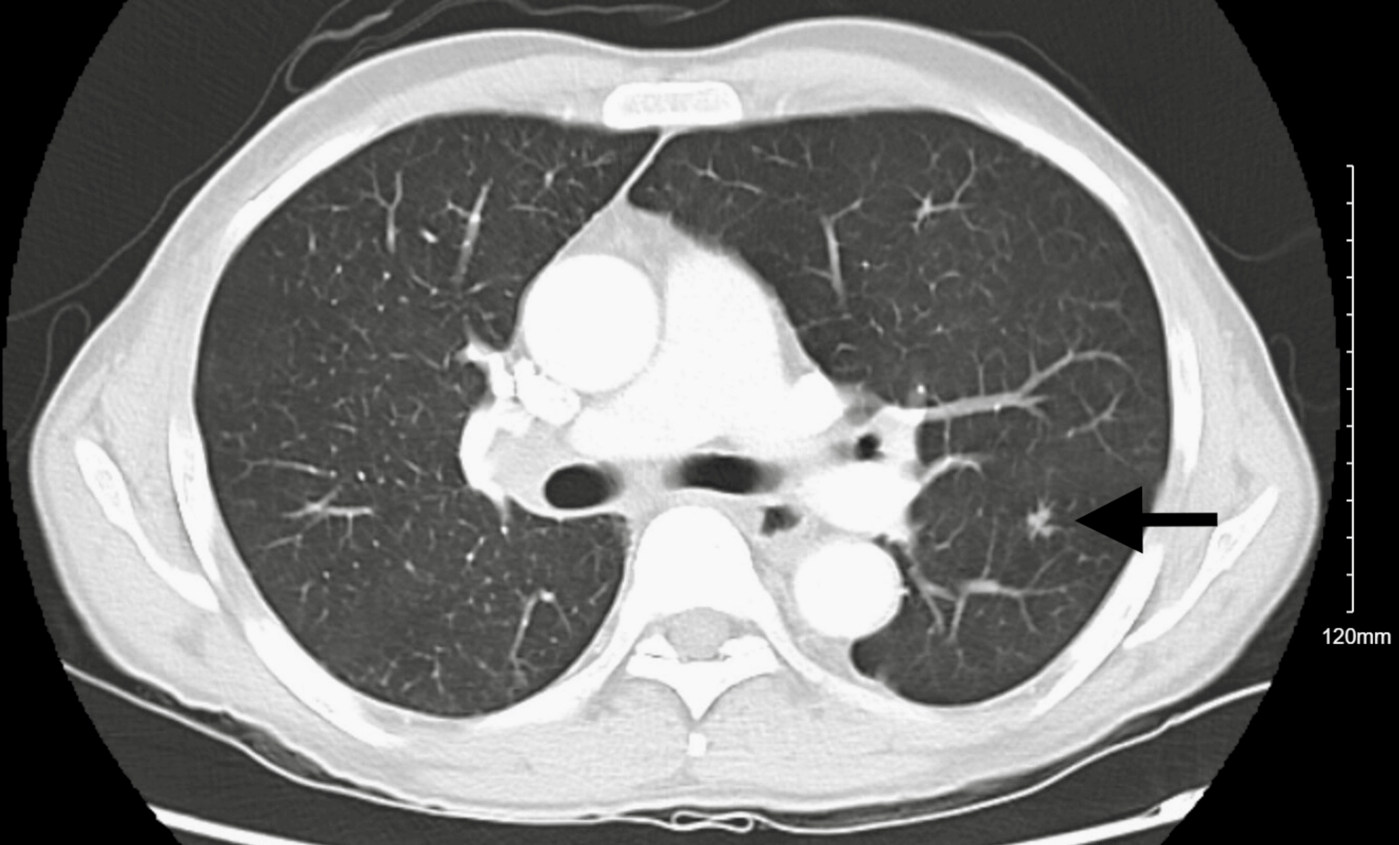
CT chest revealing tree-in-bud appearance

**Figure 2:**
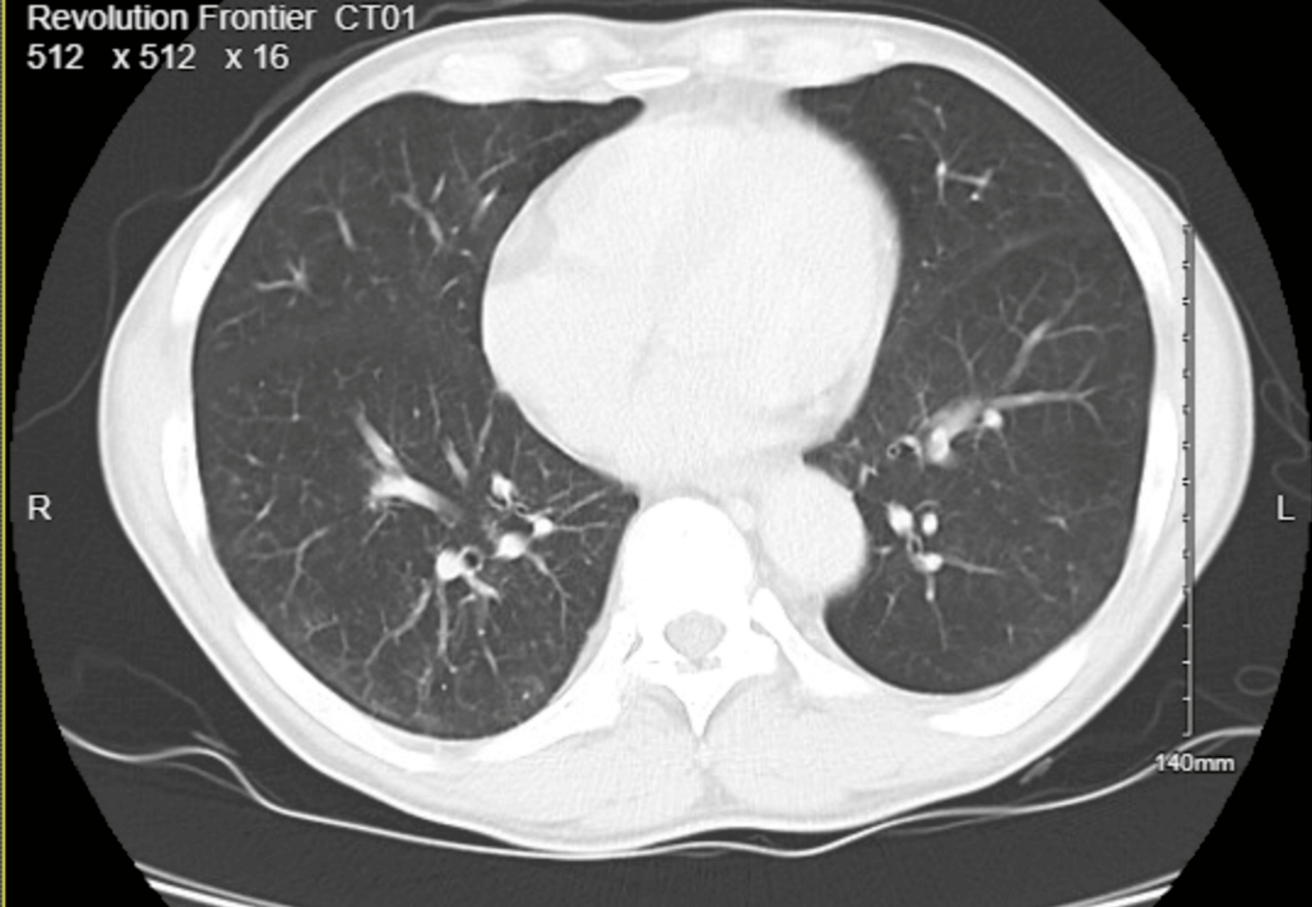
CT chest showing evidence of ground-glass abnormalities, more appreciable in the right lung

**Figure 3:**
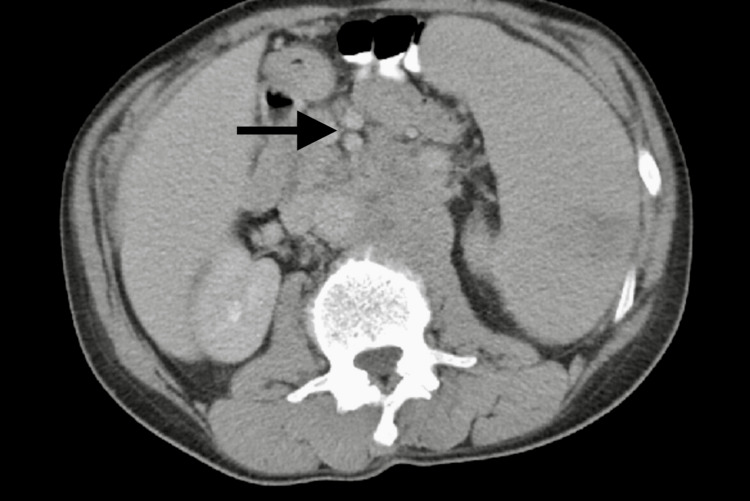
CT abdomen and pelvis showing evidence of abdominal lymphadenopathy

**Figure 4:**
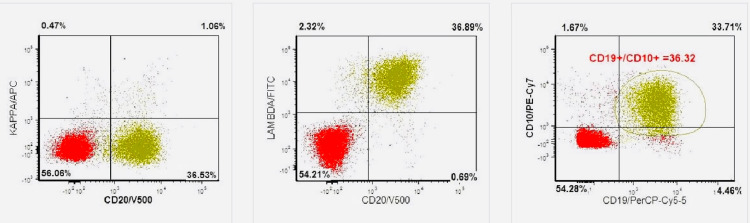
Peripheral blood flow cytometry showing the presence of monoclonal lambda CD-10 lymphoma cells (lymphoid-associated markers - moderate lambda (bright), CD19, CD20, CD22 (bright); negative myeloid-associated markers; miscellaneous markers - moderate CD10, CD38, CD45 (bright), HLA-DR)

CT chest revealed mediastinal and axillary lymphadenopathy, vague ground-glass opacities, and interstitial thickening with a tree-in-bud pattern of inflammation (Figures 1, 2). The patient was started on Levofloxacin for high suspicion of pneumonitis and was discharged due to the patient’s preferences. Abdominal imaging revealed gross retroperitoneal and periportal lymphadenopathy (Figure 3). HTLV 1/2 testing and T-cell receptor gene rearrangement testing were negative. Peripheral blood FISH (fluorescence in-situ hybridization) analysis showed evidence of high-grade B-cell lymphoma (HGBCL) with MYC (36.5%) and BCL2 (92.9%) rearrangements (Table 1). The flow cytometry confirmed the presence of monoclonal CD10-positive B-cells, one of the most common markers of diffuse large B-cell lymphoma (DLBCL) (Figure 4). After a couple of missed office appointments, the patient was called on the phone to be informed about the aggressive nature of the B-cell lymphoma. After a few weeks of non-compliance with medical advice, the patient finally agreed to a lymph node biopsy. 

**Table 1:** FISH analysis of peripheral blood showing the evidence of dual gene rearrangements of MYC and BCL2 FISH - fluorescence in-situ hybridization

Probe	% Positive Nuclei	% Control values	Signals	# Nuclei Examined
18q21 (BCL2) Kreatech (Leica BioSystems)	29.0	0-3.8	1R1G1F	200
3q27 (BCL6) Kreatech (Leica BioSystems)	0	0-3.8	1R1G1F	200
8q24 (MYC) Kreatech (Leica BioSystems)	36.5	0-3.0	1R1G1F	200

**Figure 5:**
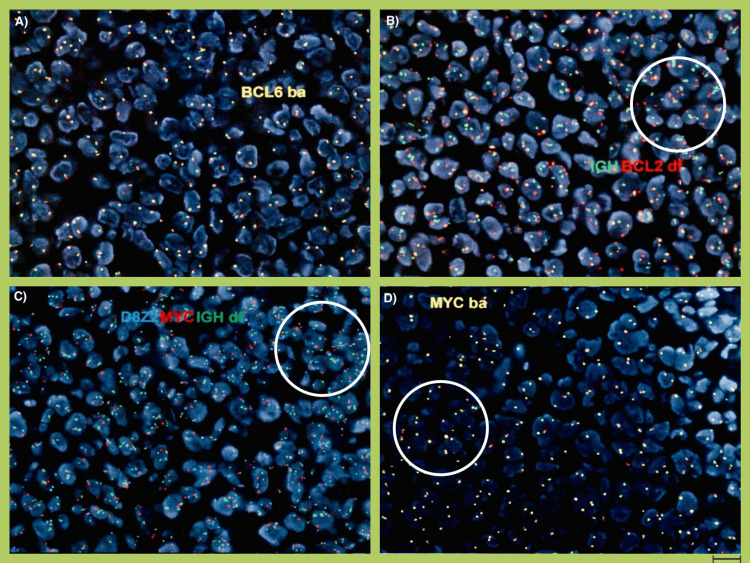
FISH analysis after lymph node biopsy: A) Normal signal pattern on a BCL6 BA probe on FISH analysis of the lymph node biopsy, showing the absence of a BCL6 rearrangement; B) Abnormal signal pattern on IGH-BCL2 DF probe (green and red, respectively) (circled area showing abnormal pattern); C) Abnormal signal pattern on a D8Z2-MYC-IGH DF (blue, red, and green, respectively) probe (circled area showing abnormal pattern); D) Abnormal signal pattern on an MYC BA probe (circled area showing an abnormal pattern) FISH - fluorescence in-situ hybridization; BA - break apart; IGH - immunoglobulin heavy chain; DF - dual fusion Magnification of 63x, scale of 10 micrometers (bottom right)

The patient was admitted for tumor lysis syndrome after an outpatient IR-guided left inguinal lymph node biopsy. The patient also received blood products during his hospital stay. Fluorescent in-situ hybridization (FISH) on lymph node biopsy confirmed the rearrangement of MYC and BCL2 genes, indicating the diagnosis of high-grade B-cell lymphoma (HGBCL) (Table 2 and Figure 5). He was also treated for neutropenic fever with anti-pseudomonal and antifungal coverage. The patient was started on R-ECHOP - Rituximab, Cyclophosphamide, Doxorubicin, Vincristine, and Prednisone. He tolerated the chemotherapy well with partial clinical response. The labs were positive for HSV 1/2 antibodies, and the patient was started on Acyclovir. The final diagnosis for the patient was double hit-lymphoma - high-grade B-cell lymphoma with MYC and BCL-2 rearrangements, a particularly aggressive form of DLBCL with an abysmal prognosis.

**Table 2:** FISH analysis of lymph node biopsy revealing dual genetic rearrangements of MYC and BCL2 in the form of abnormal signal patterns FISH - fluorescence in-situ hybridization

Probe Name	Signal Pattern	Number of Cells	Percent of Cells (%)	Total Cells	Lab Reference Range Normal/Abnormal	Normal/Abnormal
BCL6 ba	2 BCL6	190	95	200	93 - 100% / > 2%	Normal
IGH-BCL2 DF	1 IGH, 1 BCL2, 2 FUS	123	61.5	200	91 - 100% / > 2%	Abnormal
MYC-IGH-D8Z2 DF	3 MYC, 3 IGH, 2 D8Z2	21	42	50	94 - 100% / > 2%	Abnormal
MYC ba	MYC rearrangement	23	46	50	93 - 100% / > 3%	Abnormal

**Figure 6:**
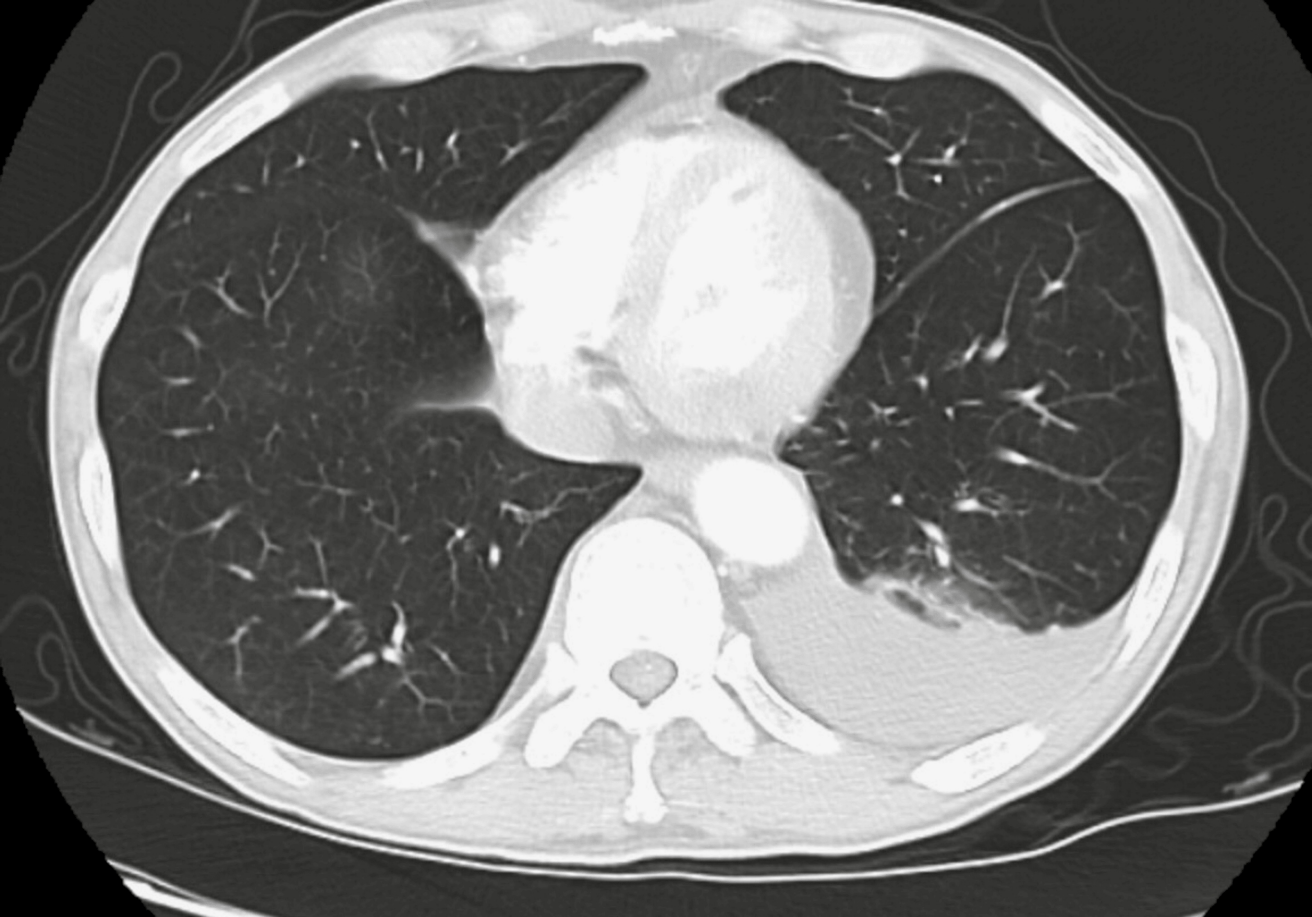
CT chest showing a large, complicated, left-sided pleural effusion

After a few weeks, the patient developed a new-onset right facial palsy, a stroke was ruled out, and the patient was treated with Acyclovir for Bell’s palsy, which was eventually switched to Valacyclovir for better compliance. He received another course of broad-spectrum coverage in the setting of neutropenic fever. He also developed a large pleural effusion (Figure 6), for which Pulmonology was consulted. A thoracentesis was performed, and 500 ml of serosanguinous fluid was removed. Pleural fluid cytology showed monoclonal lambda B-cells, about 3% of the total cell population. The patient had completed three cycles of the R-EPOCH regimen during this time. He was also kept on Sulfamethoxazole-Trimethoprim for Pneumocystis carinii prophylaxis. After being discharged home, the patient was admitted for treatment of acute pulmonary embolism and was started on Apixaban.

The patient returned to the hospital after a few days with dyspnea, and chest imaging revealed a large, left-sided pleural effusion with multiple loculations. An IR-guided chest tube was placed, and 1200 ml of serosanguinous fluid was removed. A MIST (multicenter intrapleural sepsis trial) II protocol was performed, i.e., twice daily administration of intrapleural tissue plasminogen activator and DNase separated by 12 hours for three days. Pleural fluid cytology was negative for malignant cells. The patient had received five cycles of R-EPOCH chemotherapy by then. A PET-CT was deferred until after the sixth chemotherapy cycle.

**Figure 7:**
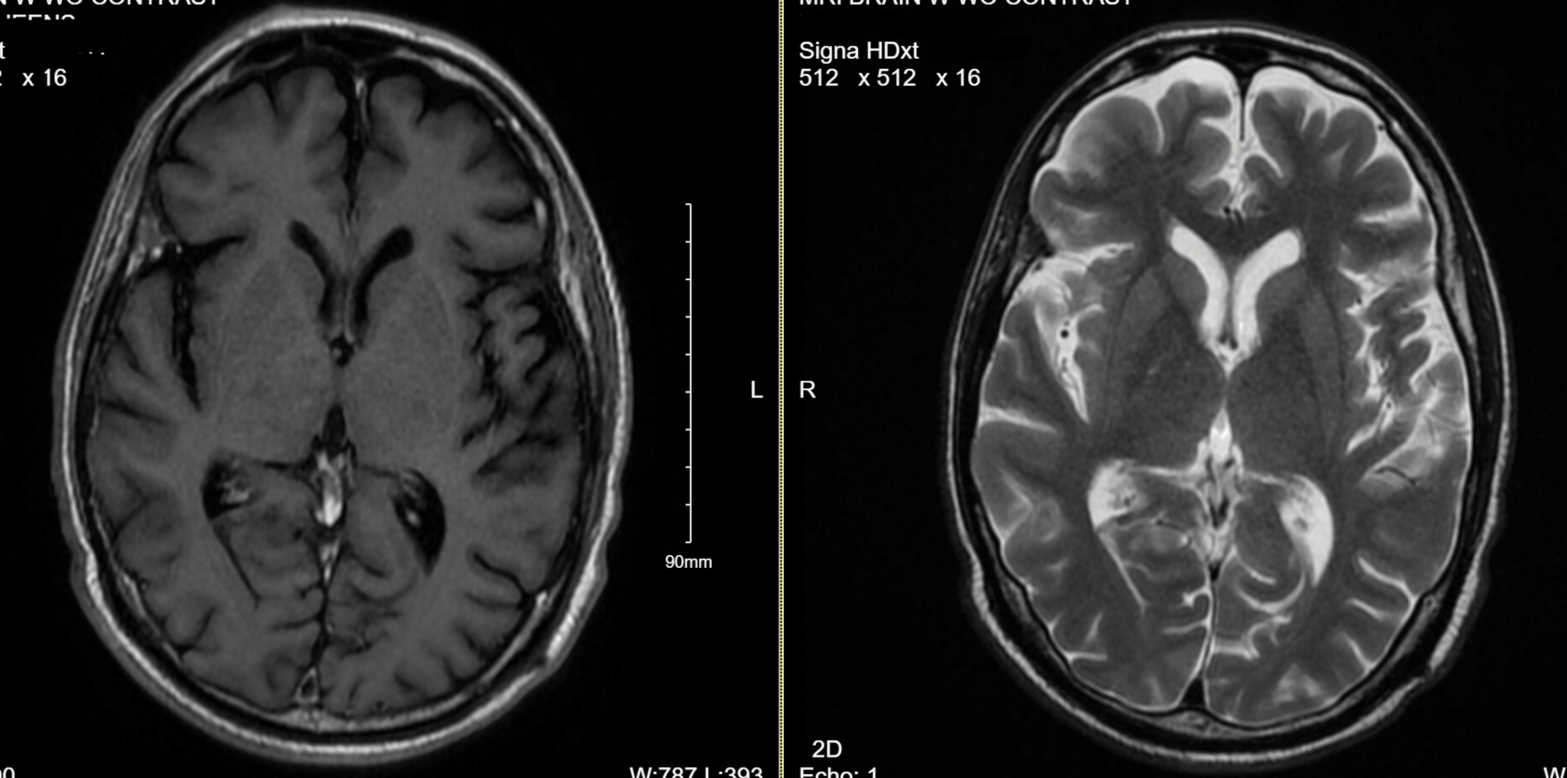
MRI brain - T1-contrast (left-hand pane) and T2 fast spin echo (right-hand pane) images that were negative for leptomeningeal enhancement

The patient, after having been discharged post-chest tube removal, presented to the ER with generalized weakness and inability to ambulate. A neurological exam was significant for the inability of the right eye to cross the midline when looking to the left, indicating right internuclear ophthalmoplegia with right eye ptosis and new-onset left facial weakness. The patient also demonstrated bilaterally weak orbicularis oculi and oris, and the presumptive diagnosis was carcinomatosis meningitis in the setting of involvement of multiple cranial nerves and negative MRI brain (Figure 7). His neurological symptoms continued to deteriorate with progressive weakness over the right side of the body, along with bilateral lower extremity weakness. A lumbar puncture confirmed the presence of leptomeningeal involvement with lymphoma, confirming the diagnosis of carcinomatous meningitis (Table 3). The patient was referred to a lymphoma center for further management and began intrathecal chemotherapy, but unfortunately, he passed away within three weeks of that.

**Table 3:** CSF findings from the lumbar puncture showing evidence of carcinomatous meningitis

Parameters	Normal range	Measured values
Appearance	Clear	Clear
Pressure (cm H_2_O)	9-18	26
WBCs (cells / mm^3^), predominant type	0-5, lymphocytes	24, lymphocytes
Glucose (mg/dL)	50-75	40
Protein (mg/dL)	15-40	48
Cytology	0-5 lymphocytes or monocytes	Positive for lymphoma cells

## Discussion

Diffuse large B-cell lymphomas (DLBCLs) can be classified as DLBCL-NOS (not otherwise specified), double-/triple-hit lymphoma, and HGBCL-NOS (not otherwise specified). We present this case of double-hit lymphoma with dual gene rearrangements in MYC and BCL2 to reiterate the necessity to consider treatment-resistant HGBCLs and their particularly aggressive nature with high mortality rates. The patients with double-hit lymphomas usually present at an advanced stage, typically have extranodal involvement, and have a high IPI (International Prognostic Index) (Table 4), which is a score developed to predict outcomes in patients with aggressive non-Hodgkin’s lymphoma based on clinical characteristics before treatment [4].

**Table 4:** Risk factors for the definition of the International Prognostic Index (IPI) ECOG - Eastern Cooperative Oncology Group; LDH - lactate dehydrogenase

Parameters	0 points	1 point
Age (years)	≤60	>60
Ann Arbor stage	Stage I or II (localized disease)	Stage III or IV (advanced disease)
ECOG performance status	0 or 1	≥2
Serum LDH	≤1× normal	>1× normal
Extranodal sites	≤1	>1

The overall incidence of double- and triple-hit lymphomas makes up 6-14% of DLBCL cases [10]. Hancock et al. performed a retrospective analysis with 31 patients and reported that three patients (10%) of the patients had CNS involvement; these patients had CNS IPI scores of >4, which indicates a poor prognosis (Table 5). Our 63-year-old male had an IPI score [11] of 5 for his age, Ann Arbor Stage III, serum lactate dehydrogenase (LDH) within normal limits three times, and with more than one extranodal site involvement, classifying him as a high-risk group with a poor prognosis. Petrich AM et al. conducted a multicenter retrospective study of 311 patients with double-hit lymphoma, which showed the median progression-free survival to be 11 months and median overall survival to be 22 months [12]. Our presented case survived for about seven months after being diagnosed with double-hit lymphoma. He died within three weeks after being diagnosed with carcinomatous meningitis despite the initiation of intrathecal chemotherapy, which compares with the review conducted by Thakkar JP et al., who reported that the median time of survival is two to four months, even with treatment [9].

**Table 5:** Risk groups of the International Prognostic Index (IPI) R-IPI – Revised IPI – provides greater discrimination among DLBCL patients with differing survival; DLBCL – diffuse large B-cell lymphoma

IPI categories	Score	Risk group	4-year overall survival, %	4-year progression-free survival, %
R-IPI	0	Very good	94	94
	1–2	Good	79	80
	3–5	Poor	55	53
IPI	0–1	Low	82	85
	2	Low-intermediate	81	80
	3	High-intermediate	49	57
	4–5	High	59	51

Double-hit lymphomas (DHL) can be diagnosed by fluorescent in-situ hybridization (FISH), which would reveal translocations of MYC (8q24) and BCL2 (18q21) or/and BCL6 (3q27) in double-hit or triple-hit lymphomas (THL) [13]. Multiple groups have compared the prognoses between the different lymphoma types and found that DHL-BCL2 and THL have similar overall survival. A few studies suggest that DHL-BCL6 is the more aggressive lymphoma [14], involves extranodal sites, and has a worse overall survival. Other studies indicate that DHL-BCL2 has a worse prognosis [14,15]. Our patient with DHL-BCL2 had an abysmal prognosis with resistance to chemotherapy and extranodal spread and died after developing carcinomatous meningitis.

Carcinomatous meningitis (CM) involves the leptomeninges with lymphoma cells. CM risk factors include the histological subtype of lymphoma, CNS-IPI, kidney and adrenal involvement, and double- and triple-hit lymphomas [16]. Kaplan et al. reviewed 63 patients with CM and reported that the most frequent symptoms were headaches, seizures, lethargy, and cranial nerve involvement [17]. It has been reported in the past that the diagnosis of CM can be challenging. It is diagnosed by a lumbar puncture, followed by flow cytometry, as it has a higher sensitivity than cytology [18]. Our patient had the risk factor of having a diagnosed double-hit lymphoma for the development of CM and had presented with features suggestive of multiple cranial nerves. Other pertinent tests for diagnosis are CSF flow studies and neuroimaging. Although leptomeningeal enhancement is one of the features of CM, our patient’s MRI did not show this feature, and he was found to have CM based on the flow cytometry studies conducted on the cerebrospinal fluid, which showed lymphoma cells positive for CD10, which is a B-cell marker. Intrathecal chemotherapy is the mainstay of treatment of CM, along with systemic chemotherapy. Conventional chemotherapy used for the intrathecal route includes methotrexate, cytarabine, and, less commonly, thiotepa [19]. Our patient was treated with intrathecal methotrexate after being diagnosed with CM; however, he survived only for three weeks after the treatment was started.

## Conclusions

HGBCLs with dual gene arrangements, also known as double-hit lymphomas, have an aggressive nature, can be resistant to strong chemotherapy regimens, and can be complicated by carcinomatous meningitis despite adequate treatment and have an inferior clinical outcome. This case report of a double-hit lymphoma with a poor clinical outcome underscores the importance and necessity of future studies to further explore the prognostic factors in double- or triple-hit lymphomas and the risk factors for carcinomatous meningitis to improve patient outcomes.
